# Supplementary figures and images for: Mathematical determination of the HIV-1 matrix shell structure and its impact on the biology of HIV-1
Source: PLoS One. 2019 Nov 12;14(11):e0224965. doi: 10.1371/journal.pone.0224965 (PMC6850549; doi:10.1371/journal.pone.0224965)

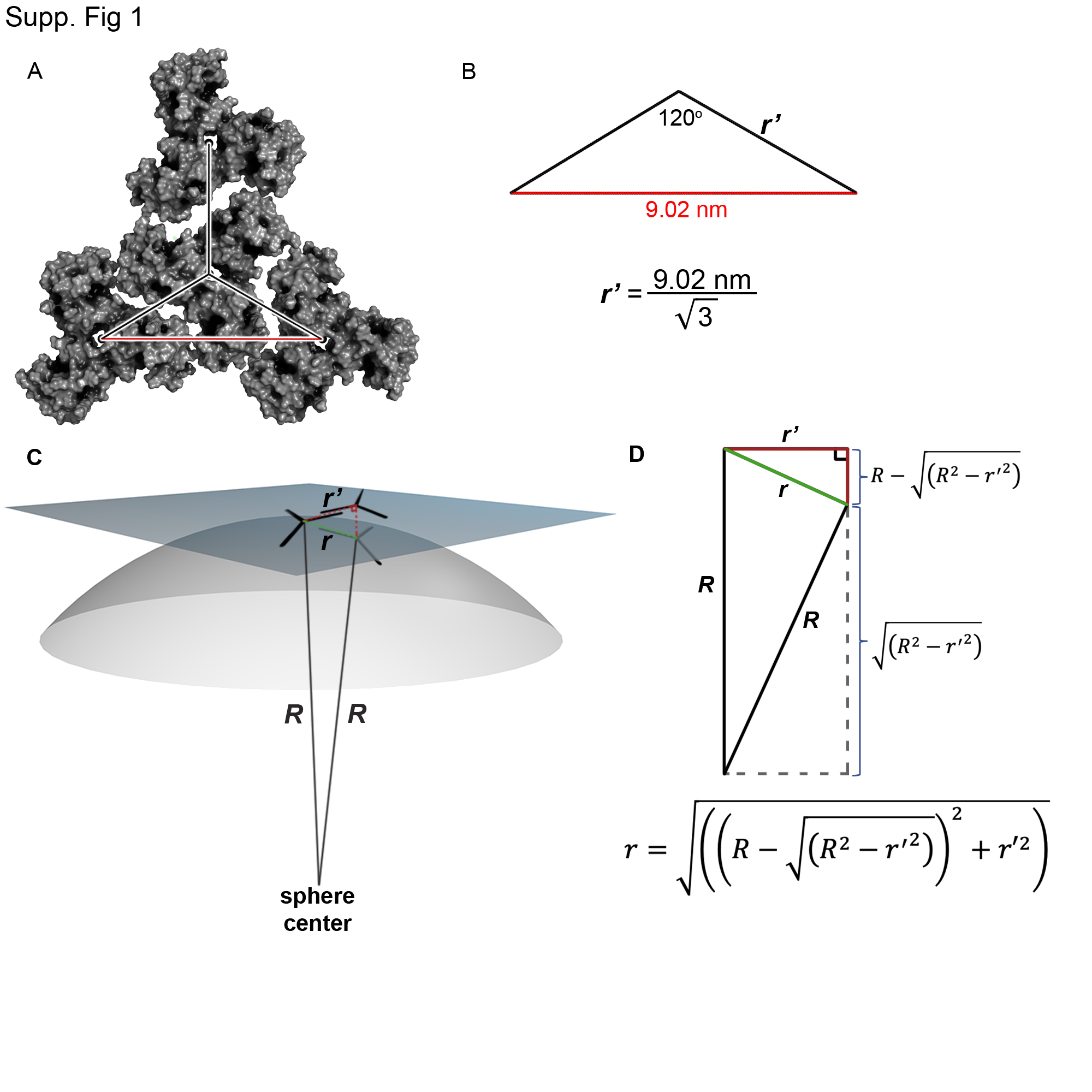

Supplement: S1 Fig — (A) The distance between the centers of mass of two adjacent trimers was obtained experimentally by Alfadhli et al. [4]. This distance is 9.02 nm and is represented by a red line. The construction process of our models required the distance between connected trimers (r’ = trimers connected by black lines). (B) The resulting triangle assumes that trimers are arranged on a flat surface. This distance can be calculated using the formula shown. (C) However, trimers are arranged on the surface of a sphere, thus we move the center of mass of trimer from a plane to the surface of the sphere while maintaining distance R. to calculate distance r. (D) Equation to calculate r and its components is shown. (TIF) [file pone.0224965.s001.tif]

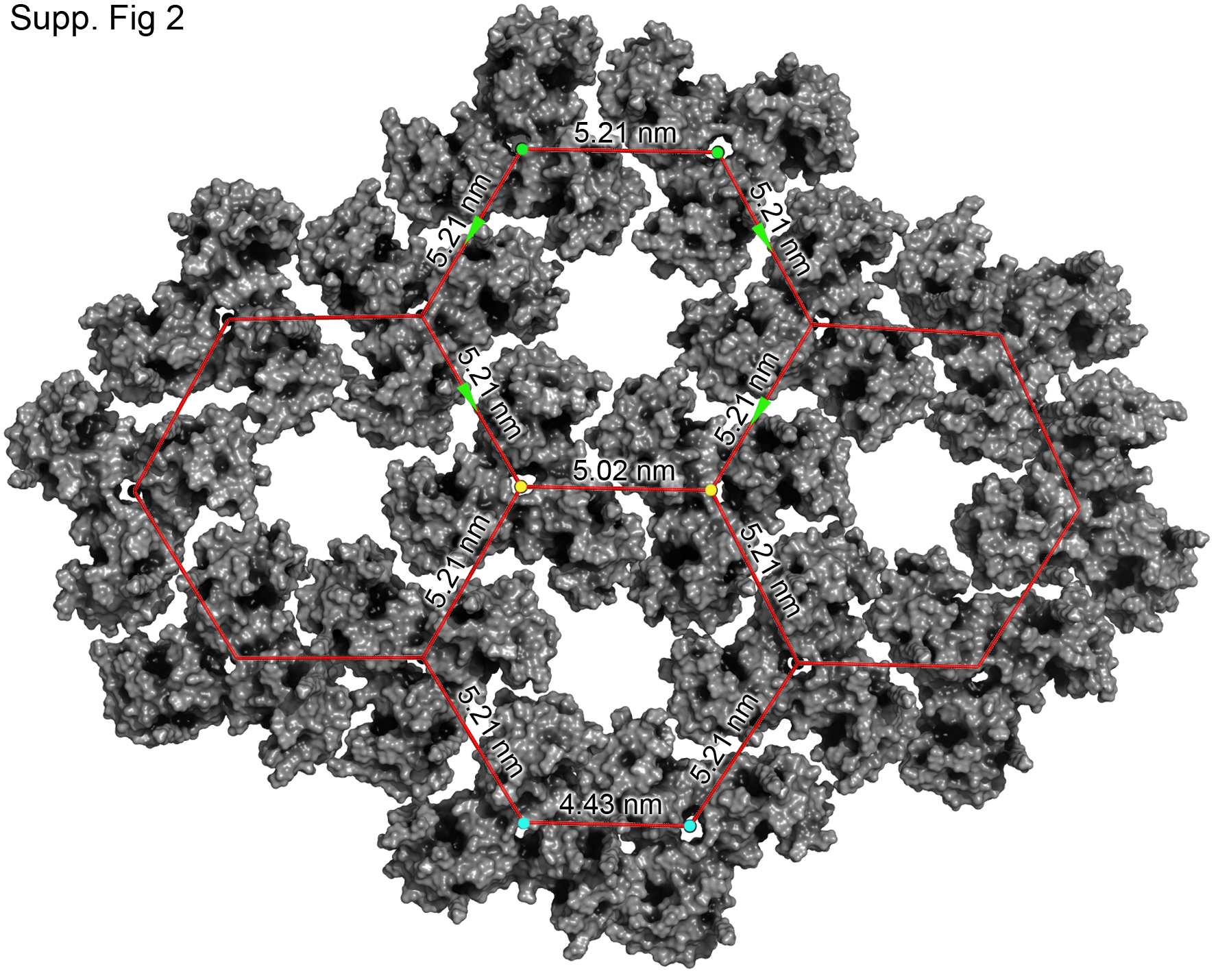

Supplement: S2 Fig — Starting from two adjacent trimers with green centers-of-mass separated by r = 5.21 nm (top trimers), we proceed with construction of the model following the direction of the green arrows to place trimers 3 and 4. Following the same procedure, trimers 5 and 6 are placed to complete the first hexagon (yellow centers-of-mass). This first hexagon is not a regular hexagon, as the final side (distance between yellow centers-of-mass) is reduced to 5.02 nm. If we proceed with creation of another adjacent hexagon, the last two trimers are separated by 4.43 nm, a distance that results in the collapse of these volumes into each other (blue centers-of-mass). Hence, it is impossible for 2 hexagons to co-exist side-by-side. The increasingly shorter ends of adjacent hexagons measure 5.02, 4.43, 3.49 nm and continues to decrease down from its original value of 5.21 nm. (TIF) [file pone.0224965.s002.tif]

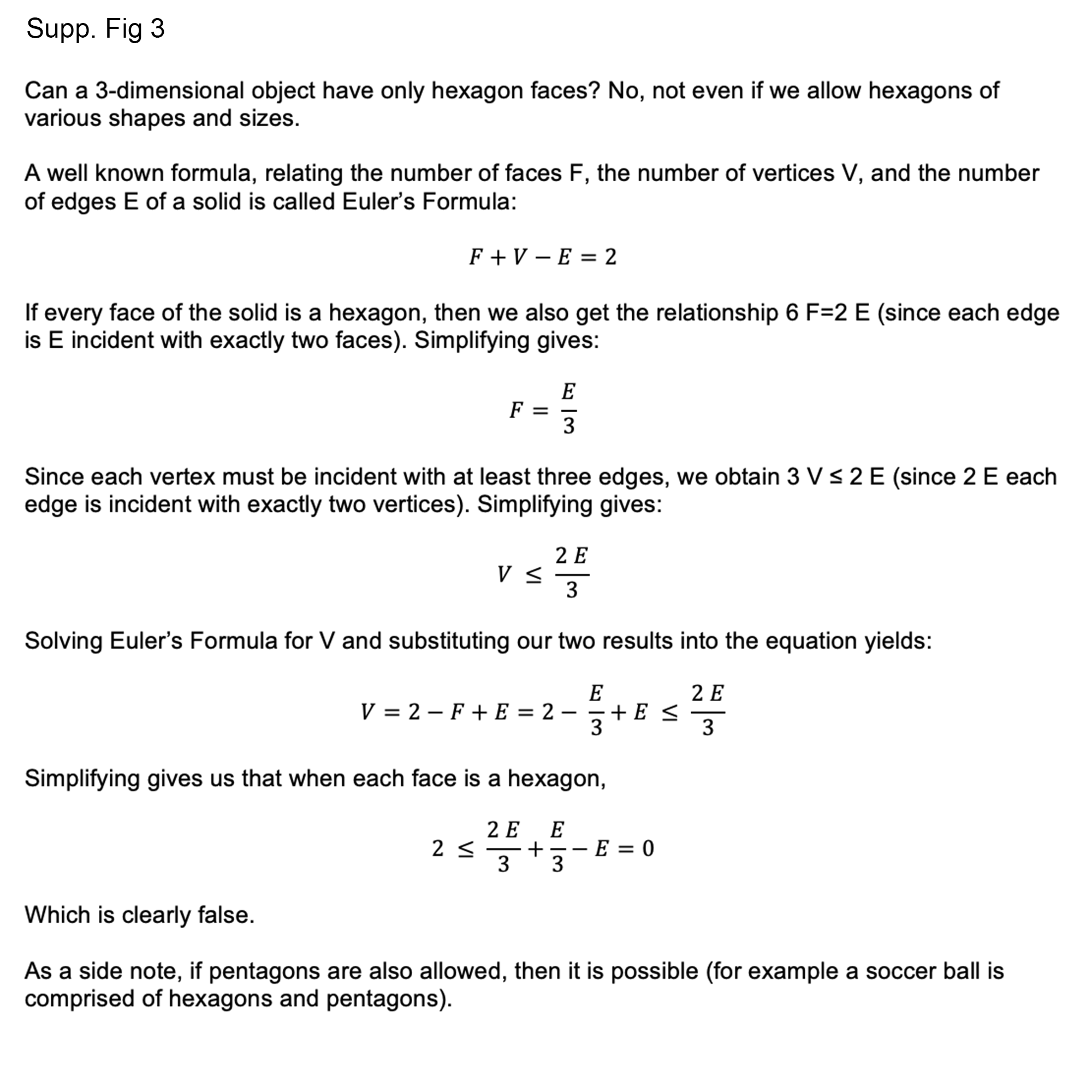

Supplement: S3 Fig — (TIF) [file pone.0224965.s003.tif]

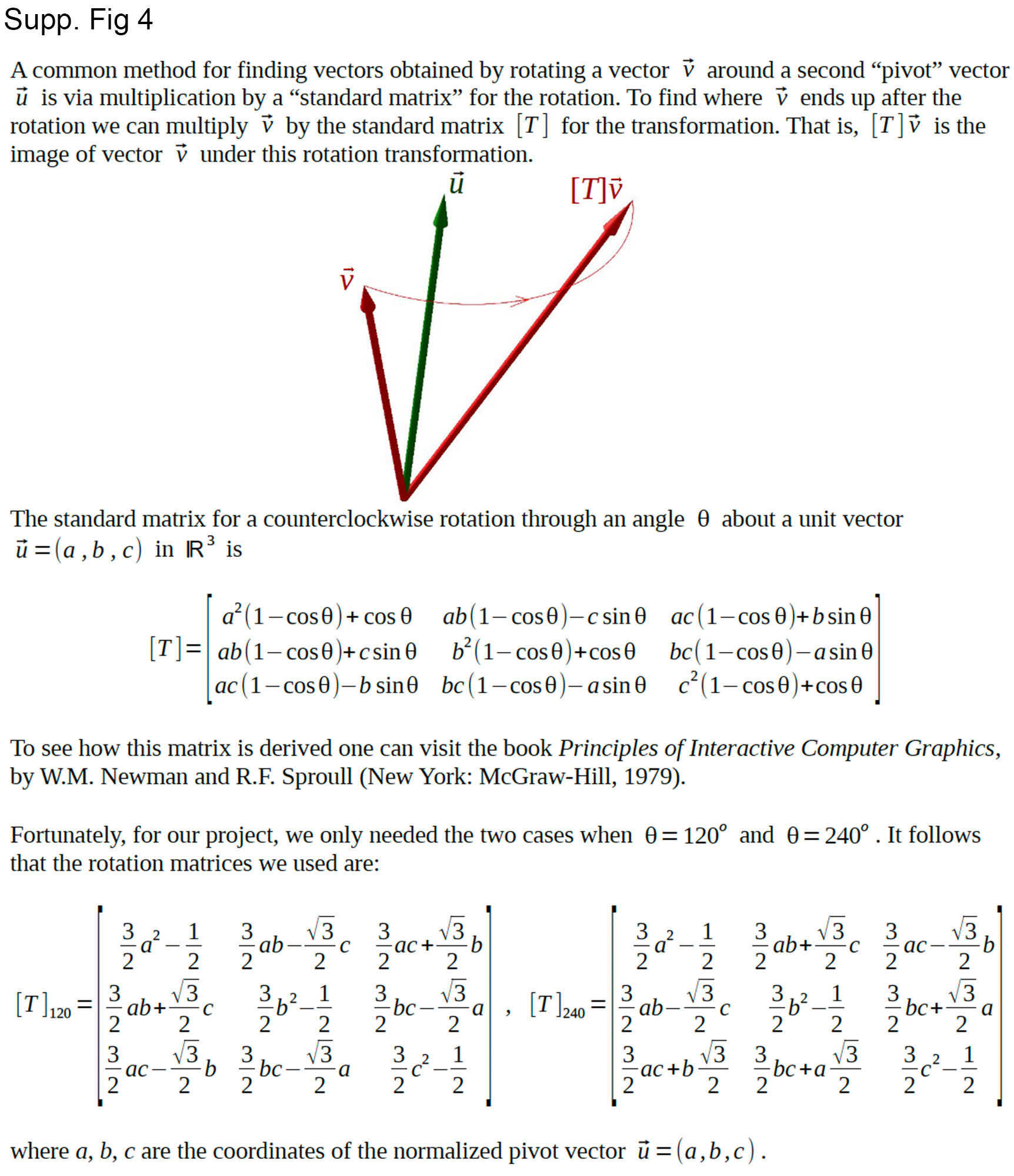

Supplement: S4 Fig — (TIF) [file pone.0224965.s004.tif]
